# The Gum–Gut Axis: Periodontitis and the Risk of Gastrointestinal Cancers

**DOI:** 10.3390/cancers15184594

**Published:** 2023-09-15

**Authors:** Giacomo Baima, Davide Giuseppe Ribaldone, Federica Romano, Mario Aimetti, Mario Romandini

**Affiliations:** 1 Department of Surgical Sciences, University of Turin, 10125 Torino, Italy; giacomo.baima@unito.it (G.B.); federica.romano@unito.it (F.R.); mario.aimetti@unito.it (M.A.); 2 Department of Medical Sciences, University of Turin, 10124 Torino, Italy; davidegiuseppe.ribaldone@unito.it; 3 Department of Periodontology, Faculty of Dentistry, University of Oslo, 0313 Oslo, Norway

**Keywords:** digestive system diseases, malignancies, neoplasms, oral bacteria, pancreatic cancer, periodontal diseases, periodontal medicine, risk factors, systemic inflammation

## Abstract

**Simple Summary:**

Periodontitis, a chronic inflammatory disease of the gums, and the oral microbiome have been recently implicated in the development of various cancers. Due to the mounting interest in the oro-intestinal axis, this review summarizes the current evidence linking periodontitis and oral bacteria to digestive tract cancers. The oral microbiome is a diverse ecosystem consisting of a variety of bacteria, some of which can move down to the gastrointestinal tract through enteral and hematogenous routes and contribute to multi-step carcinogenesis. Periodontitis and specific oral bacteria have been epidemiologically associated with an increased risk of esophageal, stomach, pancreatic, and colorectal cancers. The underlying mechanisms are still being investigated but may involve the production of carcinogenic metabolites by oral bacteria or immune evasion, as well as systemic inflammation triggered by periodontitis. These findings may have relevant implications for oral health and gastrointestinal cancer prevention, highlighting the importance of maintaining good oral hygiene and treating periodontitis.

**Abstract:**

Periodontitis has been linked to an increased risk of various chronic non-communicable diseases, including gastrointestinal cancers. Indeed, dysbiosis of the oral microbiome and immune-inflammatory pathways related to periodontitis may impact the pathophysiology of the gastrointestinal tract and its accessory organs through the so-called “gum–gut axis”. In addition to the hematogenous spread of periodontal pathogens and inflammatory cytokines, recent research suggests that oral pathobionts may translocate to the gastrointestinal tract through saliva, possibly impacting neoplastic processes in the gastrointestinal, liver, and pancreatic systems. The exact mechanisms by which oral pathogens contribute to the development of digestive tract cancers are not fully understood but may involve dysbiosis of the gut microbiome, chronic inflammation, and immune modulation/evasion, mainly through the interaction with T-helper and monocytic cells. Specifically, keystone periodontal pathogens, including *Porphyromonas gingivalis* and *Fusobacterium nucleatum*, are known to interact with the molecular hallmarks of gastrointestinal cancers, inducing genomic mutations, and promote a permissive immune microenvironment by impairing anti-tumor checkpoints. The evidence gathered here suggests a possible role of periodontitis and oral dysbiosis in the carcinogenesis of the enteral tract. The “gum–gut axis” may therefore represent a promising target for the development of strategies for the prevention and treatment of gastrointestinal cancers.

## 1. Introduction

Gastrointestinal cancers account for more than 25% of all cancers globally and 35% of related deaths. In 2020, these cancers accounted for more than 5 million incident cases and 3.5 million deaths globally [1,2]. Gastrointestinal cancer is often classified by the involved anatomical district (esophagus, stomach, liver, pancreas, colon, and rectum cancer). Although there has been considerable advancement in the timely detection of colorectal cancer (CRC), the prognosis for other gastrointestinal malignancies is often unfavorable, as they are usually detected at advanced stages [3].

Whereas traditional risk factors for these cancers include smoking, alcohol consumption, and dietary factors, emerging evidence suggests that chronic (meta)inflammation and alterations in the enteral microbiome may also play a critical role [4,5]. In recent years, growing interest has been devoted to the potential link between periodontitis, a highly prevalent inflammatory disease of the gums, and digestive tract cancers [6,7]. Additionally, the oral microbiome in general, which is a complex and diverse ecosystem of microorganisms that inhabit the mouth, has been implicated in the development of these common malignancies [8]. A great deal of epidemiologic evidence has therefore increasingly linked periodontitis/oral bacteria and gastrointestinal cancers [9,10,11], and many recent scientific works have shed new light on the potential biological underpinning of these relationships, including the direct invasion of the cancer microenvironment by oral bacteria and the systemic inflammation triggered by periodontitis.

This review aims to provide an organizing principle that summarizes the current mechanistic evidence linking periodontitis and oral bacteria to digestive tract cancers. The present overview also emphasizes the need for further research to fully understand the role of oral health in the gastrointestinal tumorigenic process, with the ultimate goal of developing novel effective interventions to prevent and possibly treat these deadly diseases.

## 2. Periodontitis and Gut Diseases: Where Is the Link?

### 2.1. What Is Periodontitis

Periodontitis is a common inflammatory disease affecting the supporting tissues around teeth, including the gingiva, periodontal ligament, and alveolar bone [12]. Currently, about 40% of all US adults suffer from periodontitis and ~11% of the world’s population is currently diagnosed with a severe form of the disease [13,14,15,16]. Periodontitis is caused by a complex interplay of (epi)genetic, environmental, and microbial factors, and it is characterized by the progressive destruction of the tooth-supporting apparatus, resulting in extensive tooth loss in its most advanced stages [17]. The disease is initiated by the accumulation of dental plaque, a biofilm of bacteria and other microorganisms that colonize the tooth surface and the adjacent soft tissues [18]. Over time, this biofilm triggers an immune response, leading to the release of pro-inflammatory cytokines and chemokines that recruit immune cells, initially neutrophils and macrophages, and later plasma and T helper 17 cells [19]. This chronic inflammation can lead to the breakdown of the periodontal tissues, resulting in the formation of periodontal pockets, and ultimately tooth loss. 

In addition to its detrimental effects on oral health, periodontitis has been associated with over 60 different systemic diseases during the past decade, including cardiovascular diseases, diabetes, respiratory affections, neurologic disorders, and digestive tract alterations, mainly inflammatory bowel disease (IBD) and cancers [20,21,22,23,24,25,26]. Although the exact causal relationships between periodontal and systemic diseases are difficult to determine due to the complexity of the underlying mechanisms, chronic low-grade bacteremia/endotoxemia and systemic inflammation caused by periodontitis are thought to play major roles [20,27]. 

### 2.2. The Emerging “Gum–Gut Axis”

In recent years, an additional pathway is garnering increasing attention, namely the enteral way. Indeed, recent studies have shown that the oral cavity and gastrointestinal tract are interconnected and can influence each other, especially from immunological and microbiological perspectives [28]. Previously, it was thought that multiple barriers maintained the separation of the oral and gut microbiota, with most salivary microbes killed by gastric acid and bile as they passed through the gastrointestinal tract. However, recent evidence has challenged this view, with even healthy individuals displaying habitual oral microbes in their feces [29]. In a study of 470 individuals, Schmidt et al. discovered that roughly one-third of intestinal bacterial strains originated from the mouth or were specialized gut subtypes of the same oral species. However, patients with IBD, bowel cancer, and rheumatoid arthritis exhibited greater oral–fecal microbial transmission than healthy controls [29].

Interestingly, emerging evidence suggests that periodontitis may influence the pathophysiology of the enteral system [30,31,32]. Several mechanisms have been put forward to explain this link. The first is the transfer of oral bacteria and their byproducts from the mouth to the digestive tract via swallowing [31]. Once in the gut, these bacteria can cause inflammation, modify the gut microbiome, and generate metabolites that facilitate pathologic processes [33,34]. Kitamoto and colleagues conducted a study in mice that demonstrated how periodontitis can cause and exacerbate gut inflammation [31]. The research demonstrated that oral pathobionts can infiltrate the gastrointestinal tract by ingestion and activate the inflammasome complex in the colon of genetically susceptible hosts. A second mechanism involves the immunological route. Indeed, in the same experimental model, periodontitis generated particular types of T helper 17 (Th17) immune cells that could move to the gut lymph nodes, where they were stimulated by oral pathobionts that translocated to the gut, ultimately causing colitis [31]. Importantly, Th17 cells from the oral cavity did not respond to antigens from gut-resident microbiota, indicating oral-specific immunity [31]. In addition, recent studies suggest the possibility of a “bidirectional effect”. Indeed, in another experiment on mice, gut translocation of oral pathobionts was shown to also exacerbate periodontitis via Th17 cells. These oral-pathobiont-responsive Th17 cells differentiated in Peyer’s patches and migrated to the head region upon oral infection. This study suggested how the promotion of periodontitis via these oral-pathobiont-responsive Th17 cells may be also mediated by the intestinal microbiome [35]. A third mechanism involves periodontitis-induced systemic inflammation, which can result in a local increase in oxidative stress, altered defense function, immune evasion, and disruption of the gut barrier function [36]. In turn, these mechanisms can lead to higher intestinal mucosa permeability and translocation of microbial products as well as inflammatory mediators into the circulation, further exacerbating chronic systemic inflammation [14,15]. 

## 3. The Oro-Intestinal Microbiome as a Carcinogen

### 3.1. Gut Microbiota in Health and Disease

The human intestinal tract harbors over 1000 species of bacteria, amounting to more than 100 trillion gut microbial cells; the majority of them reside in the colon [37]. Although a heritable component is present, studies on twins have demonstrated that environmental factors play a major role in microbiota composition [21]. In addition, within the same individual, local factors such as the availability of oxygen and nutrients, pH, and diet affect the composition and proportion of the microbiota, which indeed varies across the lumen, mucosa, and crypt–villus axis [38]. The most predominant phyla are Firmicutes (60%), Bacteroides (20%), Actinobacterium, and Enterobacteriaceae, with viruses, archaea, and fungi represented as well [39]. 

The gut microbiota is key to several features of human physiology, including immune, metabolic, and neurobehavioral traits [40]. Indeed, this meta-organism has an essential role for the fermentation of non-digestible dietary fibers, which in turn allows the growth of specialist bacteria producing short-chain fatty acids (SCFAs), amino acids, and gases [41,42]. Among SCFAs, acetate, propionate, and butyrate are the most common, and they exert several important functions, such as regulation of gluconeogenesis, training of the immune system, and control of mucosal permeability [43]. For instance, butyrate represents the principal energy source for human colonocytes, and it is also key for the maintenance of the epithelial barrier function [44]. 

There is a considerable body of evidence indicating the involvement of the gut microbiota in various health and disease states [40,43]. Notably, a plethora of mechanisms contribute to the development of diet-induced obesity and metabolic complications resulting from gut microbiota dysbiosis, including immune dysfunctions involving T cell increased avidity [45,46], altered energetic and gut hormone regulation [47], as well as the activation of proinflammatory pathways [40]. Notably, translocation of lipopolysaccharide (LPS) endotoxins across the gut barrier and their entry into the portal circulation is among the mechanisms implicated in this process [40]. These harmful microbial metabolites have the potential to affect the normal state of organs beyond the gastrointestinal tract and can have negative impacts on, for instance, the gut–brain axis and gut–liver axis [48,49]. Indeed, lower bacterial diversity, a prominent feature of dysbiosis, has been observed in the gut of patients with a wide range of chronic inflammatory diseases compared with healthy controls, including IBD [50], diabetes mellitus [51], obesity, hypertension [52], and also gastrointestinal malignancies [53].

### 3.2. Oral–Gut Dysbiosis in the Pathogenesis of Gastrointestinal Cancers

According to conservative estimates, microbes contribute to more than 15% of all cancers, resulting in an annual neoplastic burden of 1.2 million cases [54]. Regarding enteral system malignancies, *Helicobacter pylori*, a well-known colonizer of the gastric mucosa, is an example of an infectious agent associated with stomach cancer. *H. pylori* infection can cause chronic gastritis and mediate the progression to gastric atrophy, intestinal metaplasia, and ultimately gastric cancer [55]. Similarly, chronic hepatitis B and C viral infections may be key contributors to liver cancer [56], whereas certain strains of human papillomavirus (HPV) have been linked to anal and oropharyngeal cancers [57]. In the case of colon cancer, some evidence suggests that chronic infection from *Streptococcus bovis* and *Fusobacterium nucleatum* may be associated with an increased risk of the disease [58,59]. 

Beyond individual pathogens, intestinal dysbiosis, defined as an imbalance in the whole taxonomy and function of the gut microbiota, has been implicated in the pathogenesis of several types of cancer, including those of the digestive tract [60]. Indeed, dysbiosis promotes tumorigenesis through several mechanisms, including the production of pro-inflammatory metabolites such as trimethylamine N-oxide (TMAO) and LPS, the impairment of the immune response, and the induction of genotoxic stress and nitosative DNA damage [20]. Dysbiosis may also contribute to the development of pre-cancerous lesions by altering the expression of the genes involved in cell proliferation and apoptosis, such as the tumor suppressor gene TP53 [61]. Moreover, the gut microbiota influences the response to cancer therapy, since dysbiosis has been associated with a reduced efficacy and increased toxicity of chemotherapy and immunotherapy [62]. 

In recent years, periodontitis and the transfer of oral bacteria have been linked to the alteration of the microbiota composition of the enteral system, playing a relevant role in gut dysbiosis and its consequences [63]. Despite previously being considered as independent ecologic units, recent evidence is laying the ground for testing the whole oro-intestinal microbiome as a functional entity with the potential for inducing pathologic and even tumorigenic hits at both local and distant body compartments [7,34,63].

**Figure 1:**
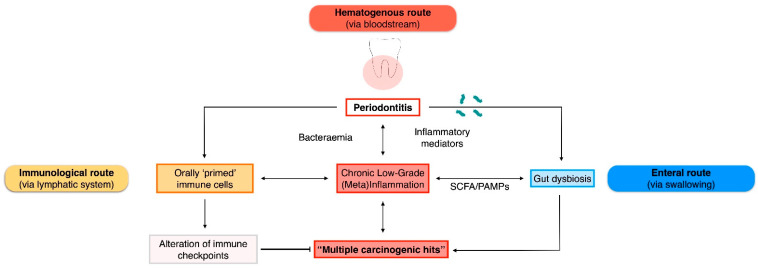
Plausible routes of interaction between periodontitis/oral bacteria and gastrointestinal malignancies. PAMPs, pathogen-associated molecular patterns; SCFA, short-chain fatty acids.

In the following paragraphs, we will present the available mechanistic evidence and interaction pathways linking periodontitis to digestive tract malignancies (Figure 1). In the absence of animal models of induced periodontitis or human trials, emphasis was given to studies exploring the virulence factors of translocating periodontal pathogens into specific cancer tissues (Table 1).

## 4. Link between Periodontitis/Oral Bacteria and Esophageal Cancers

### 4.1. Epidemiology and Risk Factors of Esophageal Cancer

Esophageal cancer represents the seventh most frequent malignancy and a top cause of cancer-related deaths worldwide [1]. The age-standardized incidence rate for esophageal cancer ranges from less than 1 to more than 50 cases per 100,000 individuals per year globally [72]. There are two main types of esophageal cancer. The first type is called esophageal adenocarcinoma (EAC), which starts in the glandular cells in the lower part of the esophagus near the stomach. EAC is the most common type of esophageal cancer in Western countries, and it is often associated with gastroesophageal reflux disease [73]. The second type of esophageal cancer is squamous cell carcinoma (ESCC), which develops in the thin, flat cells that line the upper part of the esophagus. Known risk factors for ESCC include tobacco use, alcohol consumption, obesity, and poor diet. Additional factors, such as genetic susceptibility, environmental exposures, and infection with certain pathogens, may also contribute to the development of esophageal cancer [74]. Despite advances in its treatment, the overall prognosis for esophageal cancer is still poor, with a five-year survival rate of approximately 20% [75]. Therefore, prevention strategies and early detection are critical. 

An epidemiological association between periodontitis and esophageal cancers has been described in the literature. In a large cohort study conducted in the USA, the prospective association between a history of periodontitis, edentulism, and the risk of EAC was evaluated through validated questionnaires. The study followed 98,459 women and 49,685 men for over 20 years, during which 199 incidental cases of EAC were recorded. The results indicated that a history of periodontitis was linked to a 43% increase in the risk of EAC [11].

### 4.2. Mechanistic Insights into the Perio-Esophageal Cancer Link

Research studies have shown various associations between specific oral microbiota and the risk of developing EAC or ESCC. For example, *Tannerella forsythia* and *Treponema denticola* have been linked to an increased risk of EAC, whereas symbiotic *Neisseria* spp. and *Streptococcus pneumoniae* have been associated with a lower risk [71,76,77]. In a recent study by Kawasaki et al., the presence of *T. forsythia* and *Streptococcus anginosus* in dental plaque, as well as *Aggregatibacter actinomycetemcomitans* in saliva, was associated with an increased risk of esophageal malignancies [78,79]. Additionally, a prospective study revealed higher levels of *T. forsythia* and *P. gingivalis* in oral rinse samples obtained from patients with EAC and ESCC, respectively, before their first diagnosis [80]. Additionally, *F. nucleatum* has been linked to the stage of ESCC and a worse prognosis and may be a potential biomarker for ESCC outcomes [81]. 

**Figure 2:**
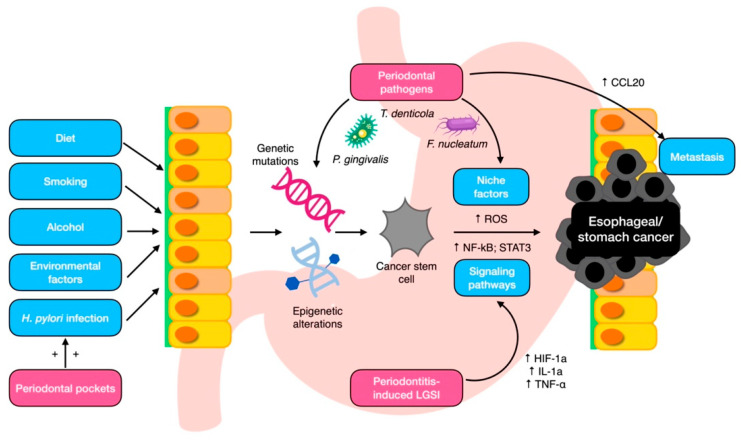
Local mechanisms for periodontal pathogen involvement in esophageal/gastric carcinogenesis. CCL20, chemokine ligand 20; HIF-1a, hypoxia-induced factor-1a; IL-1a, interleukin 1a; LGSI, low-grade systemic inflammation; NF-kB, nuclear factor kappa B; ROS, reactive oxygen species; STAT3, signal transducer and activator of transcription 3; TNF- α, tumor necrosis factor α.

The mechanisms behind the tumorigenic effects of these oral pathobionts are not yet fully understood, but it may be plausibly ascribed to some of their virulence factors (Figure 2). *P. gingivalis* is considered a putative carcinogen because of its ability to attach to keratinocytes in the gingival and upper digestive tracts, which activates the signaling pathway related to nuclear factor (NF)-κB. This activation may in turn lead to the proliferation and metastasization of ESCC cells and triggers epithelial–mesenchymal transformation through a signaling pathway activated by the transforming growth factor (TGF)-dependent Smad/YAP/TAZ [30,64,82]. Additionally, a recent enrichment analysis has suggested that *F. nucleatum* induces the chemokine CCL20, which can enhance tumor invasiveness [83]. Collectively, these findings indicate that specific oral microbiota may contribute to the development and progression of esophageal cancers through various mechanisms. Among the other species involved, *T. denticola* secretes dentilisin, a chymotrypsin-like proteinase with a high proteolytic activity, which may favor epithelial cell invasion [84,85]. 

## 5. Link between Periodontitis/Oral Bacteria and Gastric Cancers

### 5.1. Epidemiology and Risk Factors for Gastric Cancer

Gastric (or stomach) cancer represents the fifth most common malignancy worldwide and the third leading cause of cancer-related deaths [86]. The incidence and mortality rates of gastric cancer greatly varies by geographic location, with higher rates observed in Eastern Asia, Central and Eastern Europe, and South America [87]. The risk factors for gastric cancer include chronic infection with *H. pylori*, history of chronic gastritis or peptic ulcer disease, smoking, heavy alcohol consumption, a diet high in salt and preserved foods, and family history of gastric cancer [88]. Certain genetic conditions, such as Lynch syndrome, are also associated [89]. 

*H. pylori* is a Gram-negative bacterium that shows a preference for colonizing the gastric epithelium. Prolonged infection with this pathogen has been identified as the leading risk factor for the main type of gastric cancer, adenocarcinoma (GACC) [90]. In light of this, the World Health Organization has classified *H. pylori* as a class I carcinogen [91,92]. However, also other members of the gastric microbiota may also be involved in malignant transformation [93]. Indeed, gastric cancer is a histologically progressive disease that typically follows a multistep process encompassing atrophic gastritis, metaplasia, and finally malignant transformation. This progression has also been related to bacteria from the Actinobacteria, Firmicutes, Proteobacteria, and Fusobacteria phyla, which have been consistently detected in stomach biopsies derived from patients with GACC [94]. 

An epidemiological association between periodontitis and gastric cancer has also been described. Interestingly, tooth loss due to periodontitis has been significantly related with an increased risk of GAAC over a 22-to-28-year follow-up period [11]. Similarly, a large-scale study found a significant association between clinically assessed periodontitis and the incidence of gastric cancer (aHR = 1.14, 95% CI: 1.04–1.24) [95].

### 5.2. Mechanistic Insights into the Periodontitis–Gastric Cancer Link

The mechanistic link between periodontitis and gastric cancer is not fully understood, but several potential pathways have been proposed (Figure 2). First, chronic inflammation is a hallmark of both periodontitis and gastric cancer, and the systemic inflammatory response elicited in periodontitis may exacerbate the pyroptotic background that underpins gastric tumorigenesis [96,97]. This may involve the activation of pro-inflammatory mediators, such as chemokines and cytokines, which can promote tumor growth and metastasis [98]. The most relevant transcription factors implicated in the gastric-cancer-related inflammation are NF-kB and STAT3 [99]. NF-kB is activated by the toll-like receptor (TLR)–MyD88 pathway, hypoxia-inducible factor (HIF)-1a, IL-1a, and TNF-α [100], which are all pathways systemically upregulated in patients with periodontitis [101]. 

Second, the translocating oral microbiota may be a further contributor in gastric tumorigenesis [102]. Members of oral pathogenic taxa, mostly *F. nucleatum*, *Parvimonas micra*, *Peptostreptococcus stomatis*, *Slackia*, *Peptostreptococcus*, *S. anginosus*, *Parvimonas*, and *Dialister* have been robustly associated with gastric cancer [103,104]. Interestingly, these oral taxa exhibited specific niche-dependent relationships that intensified as the tumor progressed [104]. 

Third, epigenetic modifications may also be involved. Histone modifications, DNA methylation, and non-coding RNA can indeed regulate gene expression and play a critical role in gastric tumorigenesis [105]. In the in vivo study by Palioto et al., periodontitis was associated with altered DNA methylation patterns in gastric mucosa after *P. gingivalis* oral administration, suggesting a potential role for epigenetic modifications in the link between periodontitis and gastric cancer [106].

Lastly, since *H. pylori* has been shown to be a key contributor to gastric cancer development, a fourth pathway may involve the role of periodontal pockets acting as a reservoir for *H. pylori* [107]. 

## 6. Link between Periodontitis/Oral Bacteria and Pancreatic Cancers

### 6.1. Epidemiology and Risk Factors for Pancreatic Cancer

Pancreatic cancer is the seventh most common cause of cancer-related deaths worldwide, with an overall five-year survival rate of less than 10% [108]. The incidence of pancreatic cancer increases with age, with the majority of cases diagnosed in individuals over the age of 50 years. Smoking, overweight, history of chronic pancreatitis, genetic mutations such as BRCA2 and PALB2, and family history of pancreatic cancer, are all known risk factors [109]. Additionally, exposure to certain chemicals or radiation, diabetes, and *H. pylori* infection, are also considered as possible risk indicators [110]. Despite advances in treatment, the prognosis for pancreatic cancer remains poor, with most cases being diagnosed at an advanced stage.

A recent meta-analysis including eight epidemiological studies highlighted that periodontitis and tooth loss are linked to a higher risk for pancreatic malignancies, with odds ratios (ORs) of 1.7 and 1.5, respectively, after adjusting for the main common risk factors [111].

### 6.2. Mechanistic Insights into the Periodontitis–Pancreatic Cancer Link

**Figure 3:**
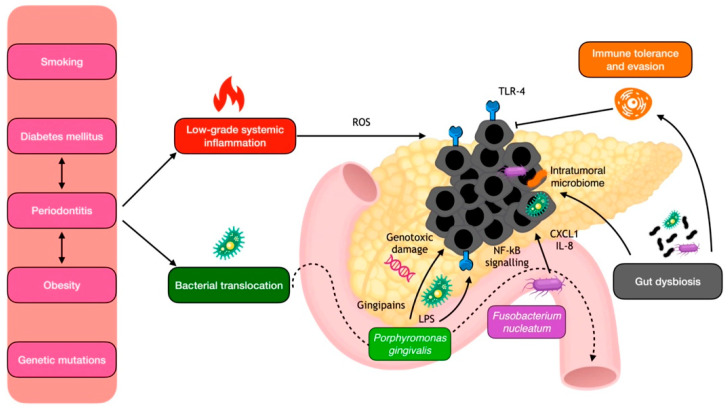
Local mechanisms for periodontal pathogen involvement in pancreatic carcinogenesis. IL-8, interleukin 8; LPS, lipopolysaccharide; NF-kB, nuclear factor kappa B; ROS, reactive oxygen species; TLR, toll-like receptors.

Diverse pathways have been proposed to explain the relationship between periodontitis and pancreatic cancer (Figure 3). One of the main mechanisms involves the chronic systemic inflammation and meta-inflammation elicited by periodontitis [112]. Indeed, leukocyte counts and C-reactive protein, which are systemic markers of chronic inflammation also elevated in periodontitis, have been associated with an increased risk of pancreatic cancer [113]. Additionally, metabolic alterations, diabetes mellitus, and obesity, which are highly associated with periodontitis, promote the chronic pancreatic spill-out of cytokines and growth factors that contribute to neoplastic development [114,115]. 

Another potential pathway is oral bacterial translocation to the pancreatic tissues, both from hematogenous and enteral routes, with consequent genotoxic damage and locally induced immune evasion. Once believed to be a sterile organ, the pancreas harbors specific bacteria that can migrate from the mouth and intestine in both health and disease states [116,117]. Notably, patients with pancreatic ductal adenocarcinoma (PDAC) displayed a distinctive periodontal pathobiont signature in both plasma, saliva, and pancreatic tumor tissues [65,118]. In a case–control study, individuals with plasma antibodies against the two main periodontal pathobionts, *P. gingivalis* and *A. actinomycetemcomitans,* had a higher risk of pancreatic cancer compared with their matched controls [118]. Additionally, a recent case–control study revealed that patients with intraductal papillary mucinous neoplasms (IPMNs) who developed invasive cancers or had high-grade dysplasia had significantly higher levels of circulating IgG reactivity to *F. nucleatum* [119]. Regarding saliva, Fan et al. conducted a study that revealed a strong association between the oral carriage of *P. gingivalis* and *A. actinomycetemcomitans* and the development of PDAC [65]. Moreover, a higher salivary-IgA-reactivity to the fibroblast activation protein-2 (Fap2) of *F. nucleatum* and *S. gordonii* was observed in high-risk IPMN cases compared with low-risk IPMN controls. Regarding the direct detection of oral bacteria in the tumor tissue, periodontitis taxa were found enriched in both the pancreatic and gut environments in PDAC patients, particularly *Gemella morbillorum* and *F. nucleatum* subsp. *vincentii* [120].

The exact mechanisms by which oral bacteria may promote pancreatic cancer are not fully understood, but it is thought that bacterial translocation may contribute to local chronic inflammation, both directly and through the disruption of the immune system. In mouse models, periodontal bacteria were indeed shown to accelerate the development of PDAC, possibly through the direct action of their virulence factors, such as *P. gingivalis* gingipains [121,122]. Moreover, TLR4 present in the gut’s mucosal lining recognizes lipopolysaccharide from *P. gingivalis*, which triggers the release of inflammatory cytokines through NF-κB signaling and the generation of reactive oxygen species (ROS), which can in turn cause DNA damage and mutations. Notably, overexpression of TLR4 has been observed in human pancreatic cancer [123]. In addition, *F. nucleatum* can increase the secretion of the cytokines GM-CSF, CXCL1, and IL-8 in PDAC cells, eliciting a phenotype associated with tumor progression [124]. In addition to their pro-inflammatory effect, periodontal pathogens may promote immune evasion, generating a tolerogenic immune program by differentially activating specific TLRs in monocytic cells [125]. Lastly, another hypothesis is that the role of periodontitis in pancreatic carcinogenesis is mediated by its induction of gut dysbiosis, which in turn may trigger immune inflammatory disfunctions in the pancreatic environment [63].

## 7. Link between Periodontitis/Oral Bacteria and Colorectal Cancers

### 7.1. Epidemiology and Risk Factors for Colorectal Cancer

CRC is the third most commonly diagnosed cancer worldwide and the second leading cause of cancer-related deaths [126]. CRC is more prevalent in developed countries, though its occurrence is rising in developing ones due to modifications in lifestyle and dietary habits. CRC incidence is age-related, with most cases occurring in individuals above 50 years [127]. Several risk factors for CRC have been found, including inherited genetic variants such as Lynch syndrome and familial adenomatous polyposis, a personal history of IBD, and family history of CRC. Additionally, lack of physical exercise, diet rich in red and processed meat, scant consumption of fruits, vegetables, fiber, and alcohol and tobacco use are modifiable risk factors [128]. 

According to a recent meta-analysis conducted by Li et al., which included seven epidemiological studies, periodontitis is associated with an increased risk of CRC (RR = 1.44, 95% CI: 1.18–1.76) [129].

### 7.2. Mechanistic Insights into the Periodontitis–CRC Link

The main mechanism proposed to explain the link between periodontitis and CRC involves the translocation of oral bacteria and their virulence factors from the oral cavity to the gut by both hematogenous and enteral routes, leading to dysbiosis and local inflammation [130]. Indeed, a metagenomic analysis of 526 samples from various populations identified seven bacteria (including *F. nucleatum*, *Porphyromonas asaccharolytica*, *P. micra*, and *Prevotella intermedia*) that were consistently enriched in CRC in different populations [131]. Specifically, in a seminal study, the relative abundance of *F. nucleatum* species considerably increased over time from intramucosal carcinoma to more advanced stages [132]. Moreover, it seems that *F. nucleatum* is linked to the CRC genetic subtype (CpG island methylator phenotype-high lesions) and tumor site (proximal tumors) [133]. 

**Figure 4:**
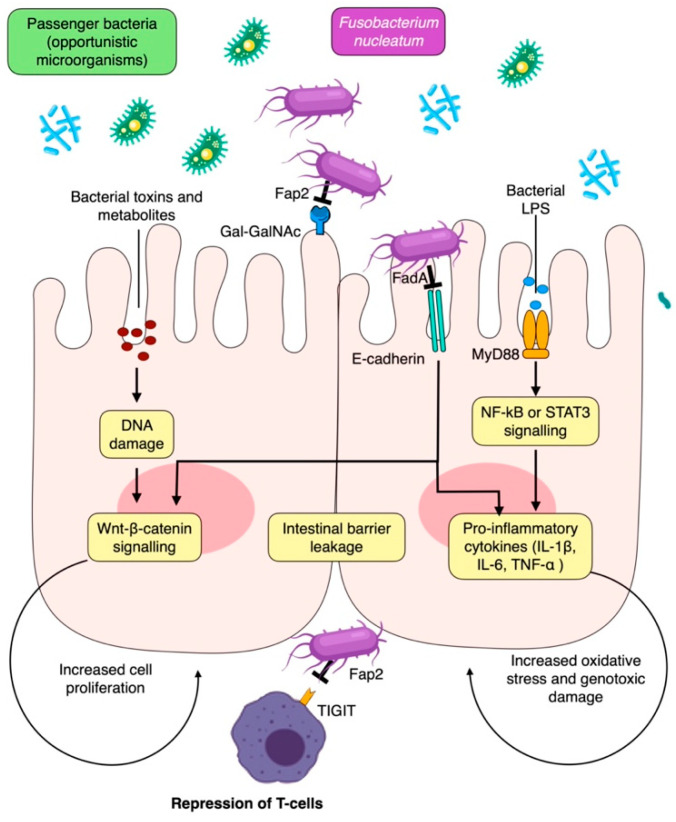
Local mechanisms for periodontal pathogen involvement in colorectal carcinogenesis. Fap2, fibroblast activation protein-2; IL-1β, interleukin 1β; LPS, lipopolysaccharide; NF-kB, nuclear factor kappa B; ROS, reactive oxygen species; STAT3, signal transducer and activator of transcription 3; TIGIT, T cell immunoreceptor with immunoglobulin and ITIM domains; TNF-α, tumor necrosis factor α.

Mechanistically, the role of *F. nucleatum* in promoting the development of CRC has been well established; it mainly does this by inducing genotoxic damage and activating various signaling pathways that contribute to tumor progression and inhibition of immune surveillance (Figure 4) [121,134,135]. *F. nucleatum* also uses its Fap2 protein to bind to Gal-GalNAc on CRC cells, facilitating colonization in the host [69]. Furthermore, *F. nucleatum* is capable of activating proinflammatory cytokines such as TNF-α, IL-6, IL-8, and IL-1β through its FadA protein, which binds to E-cadherin of intestinal epithelial cells, leading to the activation of the β-catenin and NF-κB pathways [70,121]. The bacteria’s Fap2 protein can also promote cancer progression and immune escape by binding to TIGIT receptors on NK cells and other T-lymphocyte-infiltrating tumors [121]. Lastly, *F. nucleatum* can stimulate the proliferation and invasion of CRC by activating the NF-κB pathway via TLR4 and MyD88 [136]. For these reasons, the quantification of *F. nucleatum* may even serve as a possible prognostic marker for CRC [135]. 

## 8. Link between Periodontitis/Oral Bacteria and Liver Cancers

### 8.1. Epidemiology and Risk Factors for Liver Cancer

Liver cancer is the sixth most common cancer in the world [137]. Hepatocellular carcinoma (HCC), the main type of liver malignancy, is typically asymptomatic in the first stages, thus the majority of cases are discovered when the disease is more advanced. The most frequent risk factors for HCC include exposure to aflatoxins, alcoholic cirrhosis, non-alcoholic fatty liver disease (NAFLD), and chronic hepatitis B or C virus infection. Specifically, chronic HBV/HCV infection can account for up to 80% of cases in some regions, such as in Asia and sub-Saharan Africa. Conversely, in more developed countries, the incidence of HCC is rising in parallel to the increasing prevalence of NAFLD, which is in turn linked to obesity, insulin resistance, and type 2 diabetes [138]. Additional risk factors for HCC include smoking, exposure to chemicals such as vinyl chloride and arsenic, and hereditary hemochromatosis. Unfortunately, the prognosis for HCC remains low, with less than 20% of patients with advanced-stage disease surviving at five years [139]. Prevention measures, including vaccination against HBV, screening for HCC in high-risk populations, and lifestyle modifications to reduce the risk of NAFLD, are therefore critical for the control of HCC.

In a recent systematic review, presence of periodontitis and tooth loss were associated with a broad spectrum of liver conditions, including non-alcoholic fatty liver disease, transaminase level, liver cirrhosis and also HCC, the latter with an OR of 1.34 (95% CI = 1.04–1.74) [140].

### 8.2. Mechanistic Insights into the Periodontitis–Liver Cancer Link

Researchers have shown interest in investigating the potential relationship between periodontitis and HCC, as periodontal inflammation can have an impact on circulating ROS and oxidative stress, which have been linked to the development of parenchymal cancers [141,142]. In an experimental model of ligature-induced periodontitis, increased serum ROS resulting from periodontitis was indeed shown to be detrimental to liver health by decreasing the ratio between the reduced and oxidized forms of glutathione [143]. In addition, in humans, the association between periodontitis and HCC stages has been proposed to be potentially mediated by an increase in ROS levels [144]. 

Another pathway involves the possible consequences of periodontitis-induced gut microbiota changes on the gut–liver axis [145]. Indeed, a distinctive salivary bacterial profile has been detected in subjects with HCC compared with healthy controls, the former is characterized by a higher abundance of the genera *Flifactor*, *Haemophilus*, and *Porphyromonas* [146]. This may induce a shift in the gut flora, with an enrichment of bacteria with pathogenic potential [63,147]. In turn, alterations in the gut microbiota can impact on the liver through the portal and biliary systems, thereby inducing inflammation, fibrosis, and genotoxicity, as well as activating antiapoptotic signaling pathways through various molecular patterns and metabolites (such as deoxycholic acid and LPS) [148]. These molecules may also initiate immune responses that play a role in the development of HCC [149]. In mice, the presence of *F. nucleatum* indeed decreased the diversity of the gut microbiota, increased the levels of pro-inflammatory cytokines in the serum, and reduced immune cell cytotoxicity, ultimately promoting liver metastasis [150].

Finally, a direct local effect of periodontal pathobionts in liver carcinogenesis has been proposed. In a murine model of microbially induced hepatocarcinogenesis, cytolethal distending toxins derived from *A. actinomycetemcomitans* were indeed able to significantly enhance the hepatic expression of proinflammatory genes, the growth mediators IL-6 and TGF-alpha, to increase the proliferation of HCC cells and induce genotoxic damage [67]. 

## 9. Future Research Priorities

**Figure 5:**
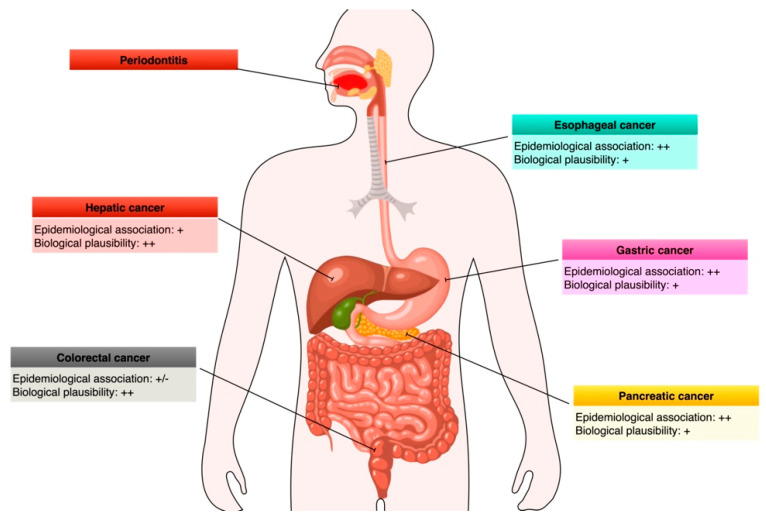
Strength of the epidemiological and mechanistic evidence linking periodontitis to the major enteral system cancers. For epidemiologic associations: +/−, inconsistent evidence; +, evidence from cross-sectional studies; ++, evidence from large prospective studies or meta-analyses. For biological plausibility: +, prevalently based on detection of oral bacteria in tumor tissues; ++, mechanistic in vitro or in vivo studies.

Despite not adopting a systematic search methodology, the present review identified key articles providing different levels of evidence linking periodontitis to gastrointestinal carcinogenesis (Figure 5). Notably, whereas preclinical studies can identify taxonomic players and mechanisms, the degree of transability to humans is uncertain. Conversely, when dealing with humans, the available studies suffer an observational design, limiting the verification of causality.

Future research on the relationship between periodontitis and gastrointestinal cancers should focus on several key areas. First, additional epidemiological studies are needed to confirm and further characterize the association between periodontitis and various types of gastrointestinal cancers. These studies should account for potential confounders, such as those from lifestyle habits [151,152], environmental contaminants [153], and other factors [152,153,154]. In particular, the role of diet, diabetes, and obesity as modulators for both the oral and gut microbiomes may be critical in mediating the susceptibility to carcinogenic stimuli [155]. Second, mechanistic studies are also still needed. Specifically, they should investigate the specific pathways and molecules involved in the translocation of oral pathogens from the oral cavity to the gastrointestinal tract and the downstream effects on immune response, inflammation, and cancer development. To determine the composition of the tumor-associated microbiota, it is recommended to utilize DNA-based techniques such as next-generation sequencing of 16S ribosomal RNA genes or whole-genome shotgun sequencing [156,157]. In addition, advances in “culturomics” and single-cell transcriptomics will allow us to understand whether this microbiome could be used as a true hallmark or is simply a bystander ascribed to the “enhanced permeability and retention effect” (i.e., greater accumulation of macromolecules in cancer tissues due to prolonged circulation and enhanced permeability) [158]. Third, studies should explore the effects of periodontitis prevention and treatment strategies in reducing the risk of gastrointestinal cancers. To this regard, diabetes mellitus may represent a relevant knot in the vicious network encompassing periodontitis, oral–gut microbiome alterations, systemic inflammation, and gastrointestinal cancers [20,159,160]. Due to the acknowledged bidirectional relationship between periodontitis and this highly prevalent metabolic disease, diabetic subjects are particularly vulnerable to this path to multi-morbidity and would benefit the most from novel targeted interventions along the gum–gut axis. Fourth, research is needed to identify biomarkers that could be used for early detection of gastrointestinal cancers, specifically in patients with periodontitis. Lastly, a major challenge is to discriminate between the role of periodontitis per se or the role that the translocating oral flora may play in the induction/progression of cancers. Whereas preclinical studies have already focused on the mechanistic aspects of the latter, in vivo models of gastrointestinal carcinogenesis and induced periodontitis should be implemented. 

## 10. Conclusions

The fight against cancer and its tremendous physical, emotional, and financial sequelae is a major priority in all public health agendas worldwide. Cancer mortality has decreased in the industrialized world in recent years as a result of considerable advancements in the understanding of the etiology of the disease, as well as in its prevention, early identification, and treatment. However, unresolved challenges remain, especially related to gastrointestinal cancers. Recent evidence suggests that periodontitis and the dysbiotic oral microbiome could play a role in the development of digestive tract malignancies. The exact mechanisms may involve dysbiosis of the gut microbiome, chronic inflammation, and direct interaction with host immune cells. Despite the rising evidence critically examined in the present review, further research is needed to unravel the underlying mechanisms and to develop effective interventions targeting the complex interplay between the pathologic oral environment and the digestive tract. Overall, the gum–gut axis represents a promising avenue for future research and public health initiatives aimed at reducing the global burden of cancer. This critical review highlighted the need for future research commitments, as well as policies aimed at reducing the exposure to risk factors from the oral cavity as part of comprehensive cancer preventive efforts. 

## Figures and Tables

**Table 1:** Mechanisms linking periodontal pathogens to gastrointestinal carcinogenesis.

Periodontal Pathogens	Main Mechanisms	Tumor Location	References
	Adhesion to keratinocytes, invasion and induction of NF-kB pathway	Esophageal	[64]
*Porphyromonas gingivalis*	Gingipain-mediated activation of the MAPK/ERK signaling pathway	Colorectal	[65,66]
	Endotoxins (LPS) induction of higher TLR4 expression	Pancreatic	
*Aggregatibacter actinomycetemcomitans*	Cytolethal distending toxin genotoxicity and activation of NF-kB pathway	Liver and colorectal	[67,68]
*Fusobacterium nucleatum*	FadA–E-cadherin interaction inducing activation of Wnt–β-catenin signaling and CRC cell proliferation Fap2–TIGIT interaction on T and NK cells inducing immune repression Fap2–Gal-GalNac interaction inducing pro-metastatic cytokines Increase the secretion of cytokines GM-CSF, CXCL1, IL-8 in cancer cells	Colorectal	[69,70]

		Pancreatic	

*Treponema denticola*	Dentilisin degradation of IL-8 and TNF-α, cleavage of pro-MMP-8 and 9	Esophageal	[71]

CRC, colorectal cancer; Fap2, fibroblast activation protein-2; GM-CSF, granulocyte-macrophage colony stimulating factor; IL-8, interleukin 8; MMP, matrix metalloproteinases; NF-kB, nuclear factor kappa B; TIGIT, T cell immunoreceptor with immunoglobulin and ITIM domains; TLR, toll-like receptor; TNF- α, tumor necrosis factor α.

## Data Availability

Not applicable.
